# 3D-Printed Dual-Channel Flow-Through Miniaturized Devices with Dual In-Channel Electrochemical Detection

**DOI:** 10.1021/acs.analchem.4c04099

**Published:** 2024-12-24

**Authors:** Miriam Chávez, Alberto Escarpa

**Affiliations:** † Department of Analytical Chemistry, Physical Chemistry and Chemical Engineering, University of Alcalá, E-28802 Madrid, Spain; ‡ Chemical Research Institute “Andrés M. Del Rio”, University of Alcalá, E-28802 Madrid, Spain

## Abstract

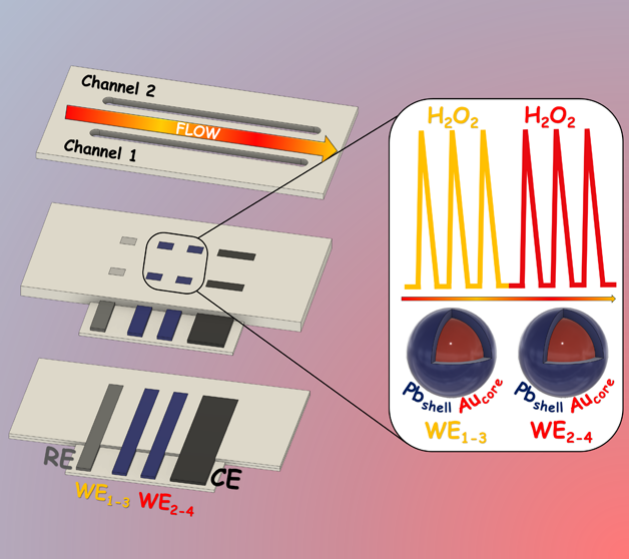

Here, we present three-dimensional-printed dual-channel flow-through miniaturized devices (3D_d_) with dual electrochemical detection (ED_d_) integrating two working electrodes each in an in-channel configuration (3D_d_-ED_d_). Prussian Blue (PB) shell-gold nanoparticles ((PB)AuNP) core-based electrochemistry was chosen for selective hydrogen peroxide determination. 3D_d_-ED_d_ devices exhibited impress stability, identical intrachannel and interchannel electrochemical performances, and excellent interdevice precision with values under 9%, revealing the reliability of the design and fabrication of the devices. 3D_d_-ED_d_ enabled long-term reliable hydrogen peroxide determination at physiological pH in Caco-2 cells under prooxidant stimulation demonstrating its outstanding electroanalytical performance. The results highlight the analytical versatility and trustworthiness of 3D-printing-based devices at miniaturized scale integrating advanced electrochemistry and its potential for real-time cell monitoring.

## Introduction

Three-dimensional (3D) printing or additive manufacturing technology fabrication of (micro)fluidics coupled electrochemical detection systems is still an emerging field particularly valuable due to their tailored design, low fabrication costs, versatility of implementation, including electrode integration and even disposability, requiring yet a tiny sample^1,2^ becoming a valuable alternative to cleanroom-based traditional glass/PDMS microfluidics.^2^

Among the variants of 3D printing technology, fused deposition modeling (FDM) is undoubtedly the most widely explored due to its easy operationality, relatively low-cost materials, and, especially, its versatility, which allows the development of flow ultraminiaturized devices with integrated electrochemical cells due to the use of conductive materials, despite its limited resolution. The FDM approach employs commercially available thermoplastic filaments that are heated to a semimolten state and extruded at a dispenser nozzle. Combining conductive and nonconductive filaments deposited layer by layer it is possible to create the 3D structure which (bio)sensing properties can be enhanced to improve its selectivity to specific analytes.^3^ Commercially available conductive filaments, such as carbon black-poly(lactic acid) (CB_PLA_), or those based on carbon fiber and graphite (Gr_PLA_) are designed to be employed in low-voltage electronic fields, where they have been successfully used directly as-printed for conductivity measurements.^4,5^ However, the current filament composition does not result in chemical sensing electrodes that are ready to use as-printed; instead, activation processes to overexpose the conductive material must be completed before its employment in analytical sensing applications. Chemical processes in conjunction with electrochemical treatment are currently positioned as one of the most common options.^6,7^ Other activation reagent-less-based approaches have also been proposed as alternatives, such as the employment of laser/plasma sources^8,9^ or a spark-discharge.^10^

Recently, it has been demonstrated that in-channel activation of CB_PLA_ electrodes by chemical and/or electrochemical means leads to ready-to-use devices.^11^ However, this procedure for activating electrodes located within the microchannels presents a disadvantage: once the channels are closed, access to them is limited, making it impossible to modify their surface after the printing process is accomplished. This disadvantage can be overcome by pausing the printing process, activating and/or modifying the electrodes, and then resuming the printing procedure until the device is closed, resulting in a one-piece structure. Such a print-pause-print (PPP) strategy allows the full customization of 3D-printed (bio)sensors.^12^

On the other hand, Prussian Blue (PB), denoted as an artificial enzyme peroxidase, is an excellent option due to its high catalytic activity and selectivity for hydrogen peroxide detection, which is an important signaling molecule in reactive oxygen species (ROS) activity.^13,14^ Compared to Pt, a typically used electrode, PB enables the detection of hydrogen peroxide by its reduction in the presence of oxygen, employing a low overpotential, and being insignificantly affected by reductant interference, thus demonstrating a much better selectivity than Pt-based sensors.^15^

PB-based electrochemical sensors can be used in many ways. The original chemical synthesis has evolved as precursors and experimental conditions are continuously being explored.^16,17^ Also, electrosynthesis strategies continue to be interesting options;^18,19^ however, the introduction of PB nanoparticles is becoming the most employed strategy,^20−23^ given the advantages offered by nanomaterials, as their large surface-to-volume ratio and increased surface activity. In this way, in the development of electrochemical sensors, simultaneous electrodeposition of PB materials and gold nanoparticles (AuNP) enhances the electrochemistry and electrocatalytic properties of the deposited PB.^24,25^ There are several cases reported in which PB is grown using different nanomaterials as scaffolds, for example, mesoporous silica,^26^ iron oxide,^27^ and gold/silver nanoparticles.^28^ Growing a PB shell using supporting nanomaterial enhances the physical stability of the hybrid nanoparticles, but the nature of the support provides further properties, such as stability for continuous detection, which has not been fully explored yet. AuNP are an excellent option for surface modification of electrochemical sensors as they present high electron density and inherent high electrical conductivity.

However, despite 3D printing’s enormous contribution to the field of biosensing, many strategies remain to be explored and exploited in 3D printing for the design and development of electrochemical (micro)fluidic systems if we take a closer look at the evolution of PDMS-based microfluidics. In this frame and inspired by our previous works, here we present 3D-printed dual-channel flow-through miniaturized devices with dual electrochemical detection integrating two PB-based working electrodes each in an in-channel configuration (3D_d_-ED_d_) for selective hydrogen peroxide detection in cells as signaling molecule of ROS. Although we have demonstrated the fabrication of fully electrochemical (micro)fluidics,^11,12^ these works are still limited to a single-channel and a single integrated electrochemical cell. However, if we want 3D printing to position itself as a real alternative to PDMS-based electrochemical (micro)fluidics, it is necessary to demonstrate that 3D printing can be used to integrate more complex electrochemical cells with tailored electrode sizes and numbers involving not only the single-channel approach. In addition, more complex electrochemistry on board needs to be demonstrated to evaluate the potency of the 3D printing technology in target applications of high significance, such as cell monitoring.

## Materials and Methods

### Reagents

Tetrachloroauric (III) acid trihydrate (HAuCl_4_·3H_2_O), calcium chloride (purity ≥97%), potassium ferrocyanide trihydrate (K_4_[Fe(CN)_6_]·3H_2_O), potassium ferricyanide (K_3_[Fe(CN)_6_]), iron chloride (III) (FeCl_3_), Nafion 5% solution, and poly(vinylpyrrolidone) (PVP, K-30, *M*_W_ = 40,000) were employed in the synthesis processes.

Sodium hydroxide pellets (NaOH), hydrogen peroxide 30% solution (H_2_O_2_), potassium chloride (KCl), Dulbecco’s modified Eagle’s medium (DMEM), phosphate-buffered saline powder (PBS), *tert*-butyl hydroperoxide (*t*-BOOH), cysteine, methionine, uric acid, and ascorbic acid were employed for various purposes along the research.

All of the above reagents were purchased from Sigma-Aldrich, are of analytical grade, and were used as received without further purification. All solutions were prepared with deionized ultrapure water produced by a Millipore system (Mili-Q).

### Electrochemical Microfluidic Device Design and Fabrication Equipment

The design of the measurement platform, including the full 3D-printed electrochemical cell, was performed with computer-aided design software (Fusion 360 Student License, Autodesk). The STL file of the design was sliced using a PrusaSlicer (Prusa, Czech Republic), and it is available upon request. Details of the printing procedure are summarized in the Supporting Section. The dimensions of the electrodes are 0.4 mm thick and 15 mm in length, but their width is slightly variable (working electrodes, reference electrode: 2.0 mm, counter electrode: 5.0 mm). Consistently, the dimensions of the grooves are 1.5 mm × 2.0 mm × 0.6 mm (working electrodes, reference electrode) and 1.5 mm × 5.0 mm × 0.6 mm (counter electrode) (width x length x height). Inlets/outlets are integrated by printing hollow cylinders with 4.0 and 1.6 mm as outer and inner diameters, respectively. Solvents and fluids were introduced inside the EMD by coupling a 32 in. ID PTFE tube (Darwin Microfluidics, France) using a short silicone tube as a connector that prevents leakage.

Once designed, the device was printed by using a Cartesian Prusa i3MK3+ instrument (Prusa, Czech Republic). Two commercial filaments were employed to manufacture most of the 3D_d_-ED_d_. Poly(ethylene terephthalate glycol) (PETg) (SmartMaterials, Spain) filament was used to print the casing, and a carbon black-filled poly(lactic acid) filament (CB_PLA_, Protopasta CDP11705, Protoplant, Canada) was employed in the construction of the electric contacts/electrodes. In addition, a cleaning filament (SmartMaterials, Spain) was used to eliminate the remaining CB_PLA_ filament from the nozzle when switching filaments. Carbon (C2180626D6, Gwent Group, U.K.) and Ag|AgCl (C2130809D5, Gwent Group, U.K.) commercial inks were employed in stencil printing.

### Electrochemical and Physicochemical Characterization

#### Electrochemical Characterization

Electrochemical experiments were carried out using a portable potentiostat/galvanostat/impedance analyzer Palmsens 4 (Palm Instruments BV, Houten, Netherlands), which presents the bipotentiostat configuration with two working electrodes, and it is equipped with PS trace software for instrument control and data acquisition. Electrochemical characterization of the 3D_d_-ED_d_ was performed using cyclic voltammetry, and hydrogen peroxide sensing was further explored through amperometry measurements. Cyclic voltammetry experiments were performed in different mediums, and depending on the objective of the experiment, a specific potential range and scan rate were chosen. Except otherwise indicated, amperometry measurements were performed under hydrodynamic conditions, using *E*_ap_ = −0.1 V (vs Ag|AgCl), and 500 μL·min^–1^ as flow rate.

#### Transmission Electron Microscopy

TEM images were obtained with a JEOL JEM 3000F instrument operating at 300 kV, which includes a microanalysis system by XEDS providing a combined morphological and chemical overview of the sample (JEOL USA Inc., Massachusetts) (ICTS-Centro Nacional de Microscopa Electrónica, Universidad Complutense de Madrid). Samples were prepared by immersing Formvar-coated Cu grids (400 mesh, Electron Microscopy Sciences) in ca. 0.1–1.0 nM nanoparticle solution for half an hour and then dunked them in water to wash away unattached material. After that, they were air-dried at room temperature.

#### Scanning Electron Microscopy

3D_d_-ED_d_ working electrode surfaces were characterized by scanning electron microscopy (SEM), using a JEOL 7600F microscope (JEOL USA, Inc., Massachusetts) (ICTS-Centro Nacional de Microscopa Electrónica, Universidad Complutense de Madrid). An acceleration voltage of 5 kV with a Schottky field-emission gun was employed for the micrographics acquisition.

#### Dynamic Light Scattering and Z Potential

The hydrodynamic size, size distribution, and Z potential of the synthesized nanoparticles were determined by dynamic light scattering (DLS) (Malvern Zetasizer Nano, ZSP) with a 633 nm He–Ne laser. The measured data are the averages of at least 20 runs. The average hydrodynamic diameter and mean Z potential of each sample were computed by using the software provided by the manufacturer. All of the measurements were performed in PBS 0.1 M (pH 7.4), and concentrations from 0.1 to 1.0 nM of the analyzed nanoparticles were tested with no significant differences observed.

#### UV–Visible Spectroscopy

Ultraviolet–visible (UV–vis) spectra to follow the evolution of nanoparticle synthesis protocols and to characterize the final material were recorded using a Jasco V-670 UV–vis–NIR spectrophotometer. Bacteria concentration was determined by measuring the optical density at 600 nm (OD_600_) using a microplate reader with a multiwell plate in a microplate reader (Synergy HTX, BioTek).

### Hybrid Au-PB Nanoparticle Synthesis

The synthesis protocol employed is an adaptation of the proposed by Yin et al.^28^ It is a two-step synthesis process, in which first potassium ferricyanide is used to introduce cyanide ions to the colloidal AuNP suspension (that contains citrate ions as capping agent, cAuNP), thus leading a preferential growth of the PB shell that can take place upon addition of the salt mixture. cAuNP were prepared using the traditional Turkevich method.^29^ Briefly, 50 mL of 1 mM HAuCl_4_ were heated to a boil barely stirring. The mixture, initially pale yellow, turned crimson once 5 mL of 38.8 mM sodium citrate was added. After 10 min of boiling, the mixture was stirred and allowed to cool to room temperature. pH of the colloidal suspension was adjusted to 10 by adding 0.1 M sodium hydroxide dropwise. cAuNP was then kept at 4 °C until further usage. Stored under these conditions, the colloidal suspension is stable for weeks.

For the next step, the previously prepared cAuNP were centrifuged at 12,500 rpm for 10 min, the supernatant was removed, and [cAuNP] was adjusted to 0.2 nM. Next, 200 μL of potassium ferricyanide (K_3_[Fe(CN)_6_]) (0.5 mM) solution was added under stirring at room temperature. After 5 min, equal amounts of 0.1 mM potassium ferrocyanide (K_4_[Fe(CN)_6_]) and 0.1 mM ferric chloride (FeCl_3_) solutions were added (200 μL, 1.25 nmol·min^–1^ addition rate). The mixture was kept under stirring for 3 h. The resulting solution was afterward centrifuged (8000 rpm, 10 min) and the precipitated Au core, PB shell hybrid nanoparticles, (PB)AuNP, were redispersed in water. Finally, the obtained nanoparticles were washed with water three times, before being dried at room temperature for 24 h.

### Preparation of PB-Based Ink from (PB)AuNP

Homemade ink containing hybrid (PB)AuNP was prepared in our lab. The simplified protocol is described as follows: 5 mg of dried (PB)AuNP powder was dispersed in 1 mL of 1 wt % Nafion solution (80:20 H_2_O/EtOH solution). Then, the suspension was gently stirred in a vortex for 2 min, before being sonicated for 30 min to ensure homogenization of the mixture. The ink is stored in darkness at 4 °C. Under these conditions, their properties are retained for several months. Before use, it is sonicated for at least 15 min to ensure proper redispersion.

Inks including other PB structures (Prussian Blue NanoCubes, PBNC, Prussian Blue NanoSpheres, PBNS, and chemically synthesized PB) instead of (PB)AuNP were also prepared. Details on these protocols can be found in the Supporting Section.

### PB-Based Sensor Fabrication

The 3D_d_-ED_d_ was prepared by combining FDM 3D printing and stencil printing technologies, introducing a pause step during the process (PPP strategy). Different parts of the electrochemical system (cell body, cell base, cell cover, channels, inlets, and outlets) were printed using PETg as a nonconductive filament, and the contacts and electrodes (working, counter, and reference) were printed with a conductive one, CB_PLA_. First, the base of the device is printed and, after loading the conductive filament, the electrical contacts are printed on the base. The insulating filament is then reinserted to print part of the body of the device, which incorporates small hollow squares on the electrical contacts to facilitate the next step. During this stage, an insulated layer that protects the electrical contacts is printed and, at the same time, helps pattern the electrodes in the following step. The 3D printing process is paused, and the final electrodes are manually customized via a stencil printing strategy employing the desired inks: commercial carbon for the counter electrodes, commercial Ag|AgCl for the reference electrodes, and homemade PB-based ink for the working electrodes. Considering that the amount of ink used is minimal, and that the bed of the printer is at a high temperature (ca. 90 °C), the drying process is very fast (a few minutes). Once the inks have dried, the printing process resumes to finish building the (pseudo)monolithic, or block, device. The surface of the working electrodes (tailored to construct the sensors) presents the multilayer structure of (PB)AuNP/C_ink_/CB_PLA_.

In the present work, batches usually include 20 devices. After manufacture, the 3D_d_-ED_d_ is stored inside plastic boxes in a desiccator at a controlled temperature (40 °C) and in the presence of silica gel as a desiccant capable of capturing a large amount of moisture to prevent deterioration. Under these conditions, the electrochemical performance of the devices remains intact for at least one month.

### Caco-2 Cell Line and Culture Medium

The Caco-2 cells were obtained from the European Collection of Authenticated Cell Cultures (ATCCHBT37 Lot. no. 7004614, LGC). They were kept in DMEMF12 medium supplemented with 10% heat-inactivated FBS, 100 units·mL^–1^ penicillin, and 100 μg·mL^–1^ streptomycin. Cultures were seeded into flasks containing supplemented medium at 37 °C under a humidified atmosphere (5% CO_2_ and 95% air). For the experiments, Caco-2 cells were subcultured in 24-well plates at a seeding density of 10 × 10^4^ cells·cm^–2^ per well. Under those conditions, Caco-2 cells reach full confluence after approximately 4 days.^30^ ROS secretion, in terms of H_2_O_2_, was determined by flow amperometry, after subjecting the cells to different experimental conditions: the amount naturally produced ROS as a function of time, and after undergoing oxidative stress with bacteria (*Escherichia coli*, *E. coli*), and a prooxidant agent such as *tert*-Butyl hydroperoxide (*t*-BOOH). We evaluate the effect of a fixed *E. coli* concentration (CFU·mL^–1^ = 6.7 × 10^4^) on the current measure at *E*_ap_ = −0.1 V over time (0 to 300 min).^31^ Concerning *t*-BOOH, we study the dose and exposure time affecting ROS generation. Electrochemical H_2_O_2_ measurement levels were made by a fluorescence assay after stressed Caco-2 cells with *t*-BOOH in the same conditions.^32^ This procedure is based on the addition of 2,7-dichlorofluorescein (DCFH-DA), which becomes DCFH after being oxidized by intracellular oxidants and emits fluorescence. The assay was performed as follows: 10 μM DCFH-DA was added to each well for 30 min (37 °C), and after that time, the cells were washed twice with DMEM. A fluorescent microplate reader was employed to record the signals using an excitation wavelength of 485 nm and an emission wavelength of 530 nm.

During the experiments, controls were always evaluated. In all cases, the corresponding controls presented the same composition of the medium studied without contact with the cell cultures.

## Results and Discussion

### 3D-Printed Dual-Channel Flow-Through Miniaturized Devices with Dual In-Channel Electrochemical Detection: Design and Fabrication

**Figure 1:**
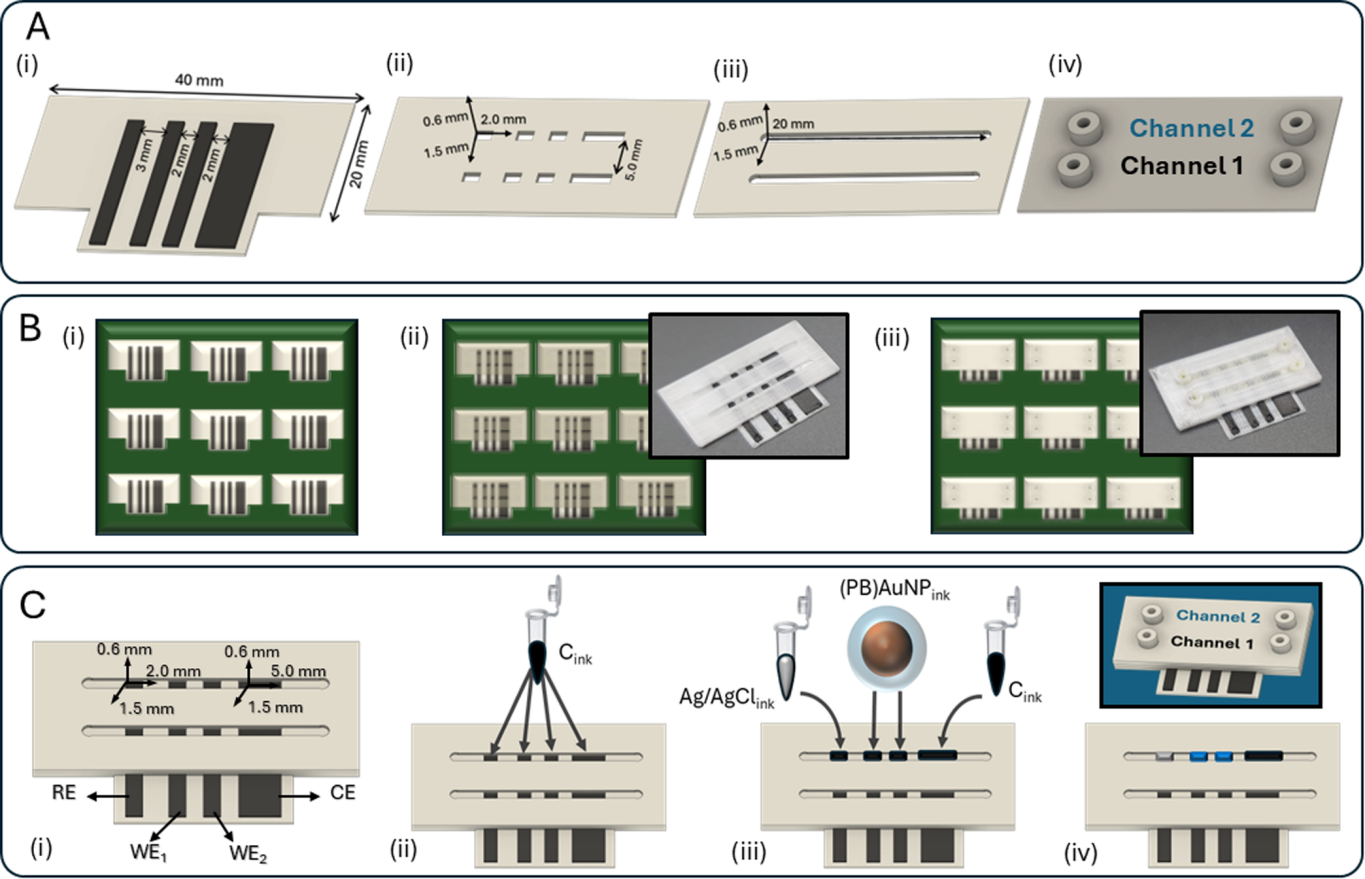
(A) Design of the 3D_d_-ED_d_ including (i) connectors/electrodes, (ii) grooves, and (iii) channel dimensions and (iv) cover with the inlets and outlets. (B) Printing sheet illustration showing the PPP process: (i) platform base, connectors/electrodes, and grooves printing, (ii) pause for stencil printing, and (iii) channel, cover, and inlet/outlet printing. Insets (ii, iii): photos of the manufactured devices. (C) (i) Details of the cell in-channel cell configuration, including dimension of the electrodes, and schematics for the electrode stencil printing process: (ii) a first layer of commercial carbon ink is applied on the 3D-printed connectors; (iii) Ag|AgCl, (PB)AuNP, and carbon inks deposited as a second layer; and (iv) electrodes appearance before restarting printing process. Inset: scheme of the complete block 3D_d_-ED_d_.

The design and fabrication of 3D_d_-ED_d_ is shown in Figure 1 (see also Figure S1).

It implies the fabrication of one electrochemical cell (Figure 1A,i-ii) through two parallel channels (Figure 1A,iii-iv) to simplify device manufacturing by integrating two pairs of working electrodes in both channels (WE_1–2_^ch1^; WE_3–4_^ch2^). To this end, it is essential to assess that the four electrode surfaces to be used as working electrodes, as well as those to be used as reference and counter electrodes, exhibit identical electrochemical behavior.

The complete fabrication process is summarized in Figure 1B: First, a nonconductive PETg base is printed, and conductive CB_PLA_ connectors are then incorporated. The printing process then continued with PETg to lift small rectangular grooves.

Electrochemical cell design was carefully considered (Figure 1C, i). Inside the cell, in both channels, the reference electrode is the first one to be reached by the solution as it enters and flows through the channel, to prevent potential displacement due to interfering products generated in the working or counter electrodes. Then, two working electrodes are placed, and finally, the counter electrode ends the electrical circuit. Their dimensions, 2.0 mm × 15.0 mm × 0.4 mm (working electrodes, reference electrode) and 5.0 mm × 15.0 mm × 0.4 mm (counter electrode) (width × length × height), and pitch separation, have also been optimized to improve the electrochemical performance. The counter electrode had larger dimensions than those of the working electrodes for accurate current measurements, as current flows between the WEs and the CE, and the total surface area of the last one must be larger so that it will not be a limiting factor in the kinetics of the electrochemical processes. On the other hand, the separation between the channels (5.0 mm) has been optimized so that the printing resolution was efficient, but without the amplified electrode length, it was translated into an excessive increase in electrical resistance.

A pause in the printing process is included at this point to perform a stencil printing modification of the different electrodes with high throughput.

Electrode modification was carried out as follows (Figure 1C, ii-iii): a relatively thicker layer (ca. 0.4 mm) of carbon ink base is included in all of the grooves. Then, a thinner PB-based ink layer (ca. 0.1 mm) is stenciled on the working electrodes and Ag|AgCl and carbon inks on the reference and counter electrodes, respectively. Finally, the 3D printing process is continued to perform microchannel fabrication, allowing us to obtain a leak-free block 3D_d_-ED_d_ (Figure 1C, iv).

### PB(AuNP): Electrochemical Characterization

Then, PB electrochemistry was carefully studied on board. Several options were explored to integrate the PB efficiently. Although widely reported, the electrochemical synthesis of PB in the working electrode surface was directly discarded, as it would be necessary to perform the electrodeposition process in the printing bed, introducing external pin connectors, thus considerably increasing the occurrence of failures during the 3D printing second step. Furthermore, electroplating must be performed individually on each 3D_d_-ED_d_, and this process should take at least several minutes to obtain optimal results. As an alternative, we explored the manufacturing of customized conductive inks based on PB nanoparticles (see the Supporting Information for details): PBNC, PBNS, and (PB)AuNP. Chemical PB synthesis was also explored. Specifically, (PB)AuNP has proven to be useful in specific applications, such as surface-enhanced resonance Raman scattering,^28^ but has not been used to date in the manufacture of low-cost electrochemical sensors.

**Figure 2:**
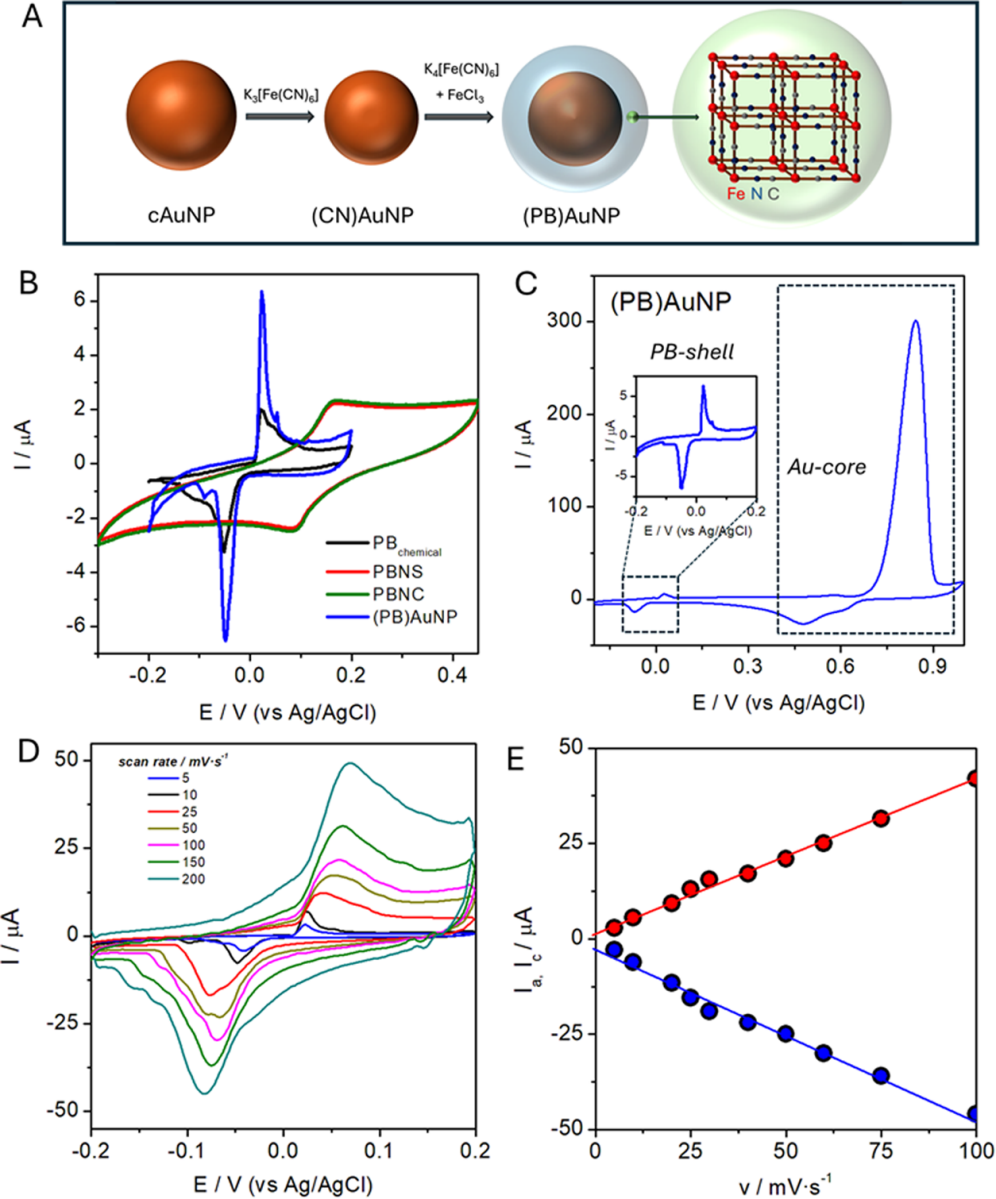
(A) (PB)AuNP synthesis scheme. (B) Cyclic voltammograms of PB-based inks and chemically deposited PB tested during the sensor fabrication (0.1 M KCl/PBS (pH 6.6), scan rate: 10 mV·s^–1^). (C) Extended cyclic voltammogram of (PB)AuNP/C_ink_/CB_PLA_, evidencing the Au core, and the PB shell, as PW/PB redox couple (0.1 M KCl/PBS (pH 6.6), scan rate: 10 mV·s^–1^). (D) Cyclic voltammograms of PB(AuNP)/C_ink_/CB_PLA_ at different scan rates (0.1 M KCl/PBS (pH 6.6)). (E) Linear variation of the anodic and cathodic currents vs the scan rate.

The selected nanoparticles were synthesized following different protocols, and after their isolation, they were treated to prepare the conductive PB-based inks. (PB)AuNP could be obtained at room temperature using a quite simple procedure (Figure 2A). These hybrid core–shell nanoparticles were synthesized from cAuNP (AuNP of ca. 20 nm stabilized using citrate anions as the capping agent). They are subjected to etching with potassium ferricyanide, slightly reducing their size and modifying the nature of their surface, being protected by cyanide anions after this step ((CN)AuNP). At these conditions, the simultaneous addition of potassium ferrocyanide and ferric chloride in equimolar concentration causes the preferential growth of a PB shell around the AuNP surface. Alternative PB nanoparticles, PBNC and PBNS, were prepared using solvothermal methods (Supporting Section).

After their synthesis, the three PB nanoparticles described above were used to prepare homemade PB-based inks, which were then stenciled onto the working electrodes. On the other hand, the chemical PB was prepared directly on the electrode surface. To elucidate which one of the proposed PB surfaces exhibits the best electrochemical performance, the explored options were compared based on the analysis of the oxidation/reduction peaks of the cyclic voltammograms of the Prussian White/Prussian Blue (PW/PB) redox couple (Figure 2B),^33^ being evidence that the nature of the PB employed to prepare each of the customized inks results in quite different voltammograms, but in all of the cases, the reversible redox couple is detected in the potential range between −0.2 and 0.2 V (0.1 M KCl/PBS, pH 6.6).

The electrochemical parameters obtained from the analysis of these voltammograms, gathered in Table S1, allow us to be unequivocally in favor of (PB)AuNP to prepare our tailored working electrodes. (PB)AuNP anodic and cathodic peaks present the lowest peak-to-peak separation (0.077 ± 0.004 V), as well as the closest to zero half-wave potential (−0.015 ± 0.001 V). The measured oxidative charge (20.5 ± 1.0 μC) is more than twice the observed for the chemically deposited PB (9.1 ± 0.6 μC), and compared to PBNC and PBNS inks (Δ*E*_p_(V) = 0.243 ± 0.013, 0.234 ± 0.015, and *E*_1/2_(V) 0.045 ± 0.005, 0.048 ± 0.002, respectively), the half-wave potential is closer to zero and the peak separation is one-third of these. This is essential for our purpose, as PB was selected for its well-known selectivity toward hydrogen peroxide detection. By applying a potential close to zero, currents related to the reduction of oxygen and other common interferents present in a biological medium are avoided, ensuring selective detection.

Further analysis of the redox couple peaks shown in Figure 2B confirms our decision in favor of (PB)AuNP. From the oxidation peak, we calculate the amount of electroactive PB. Although the calculated charge for the inks based on PBNC and PBNS (26.1 ± 0.8 and 26.0 ± 1.0 μC, respectively) are slightly higher than the estimated for (PB)AuNP (20.5 ± 1.0 μC), these values must be considered together with the rest of the electrochemical parameters. Such a charge value indicates that a significant amount of active PB is present on the working electrode surface, which is suitable for its electrocatalytic application. These results would point to that the presence of PB film on an Au core leads to a better organization of its structure, which is reflected in the shape of the cyclic voltammogram, and position this synthesis as the best option from the point of view of electrocatalysis.^24^ The width of the peaks must also be considered: (PB)AuNP ink exhibits sharp peaks (oxidation peak width of 0.022 ± 0.001 V), indicating a fast and efficient electrochemical reaction.

The extension of the potential window from −0.2 to 1.0 V provides further information about surface composition (Figure 2C). In addition to the narrow peaks attributed to PW/PB (inset), a broad oxidation peak (ca. 0.7 to 0.9 V), and a distinct reduction peak with a maximum at ca. 0.5 V are indicative of the oxidation and subsequent reduction of gold oxide (AuOx) of AuNPs.^34^

Besides, electron transfer of the redox couple PW/PB was investigated by scan rate studies (Figure 2D,E). At the lowest scan rates, both anodic and cathodic currents vary linearly with the scan rate, as expected for a diffusion-controlled process.^35^ The narrow shape of the peaks evidences that the PB-based NP keeps its fast diffusion electron transfer rate and a reduced resistance within the electrode, even when it is in the form of conductive ink. Furthermore, peak-to-peak separation is almost constant at scan rates below 0.075 V·s^–1^, demonstrating an efficient charge-transfer kinetics process in that range, while at higher scan rates, such parameter increases linearly with the logarithm of cycling rate (results are not shown). Both anodic and cathodic processes exhibit correct regression equations of *I*_a_ (μA) = (0.43 ± 0.01) v (mV·s^–1^)+ (1.7 ± 0.1), and *I*_c_ (μA) = (−0.46 ± 0.02) v (mV·s^–1^) – (2.5 ± 0.5), respectively, with good linearity (*R*^2^ = 0.990).

### PB(AuNP)-Based Inks Characterization

**Figure 3:**
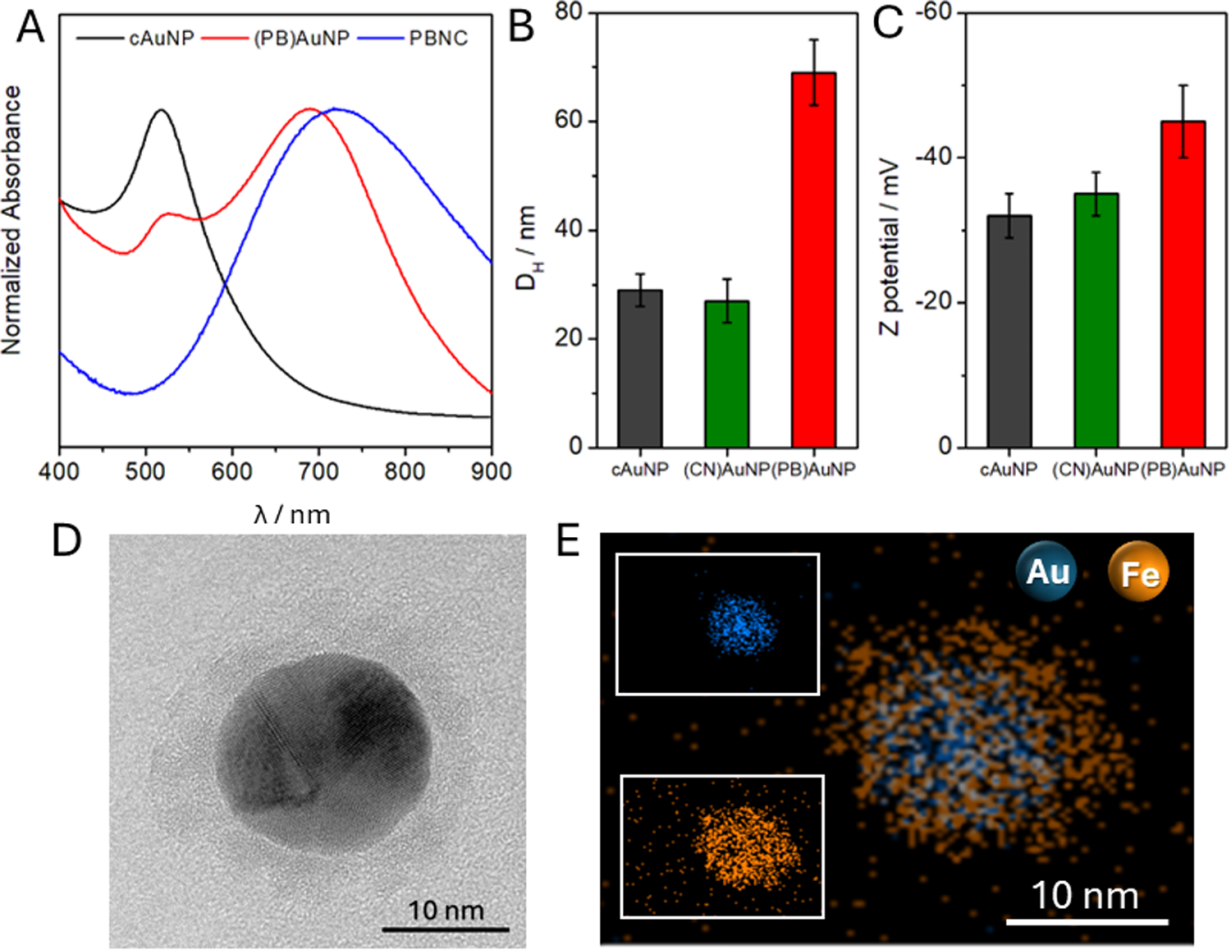
(A) UV–vis spectra of cAuNP (black), (PB)AuNP (red), and PBNC (blue) recorded from 400 to 900 nm. Average (B) hydrodynamic diameter, and (C) Z potential value for the different steps during (PB)AuNP synthesis (recorded in 0.1 M PBS, pH 7.4). Values in graphics bars of (B, C) are expressed as mean values ± standard deviation (*n* = 3 each). (D) TEM image of an isolated (PB)AuNP. (E) SEM-EDX elemental mapping of (PB)AuNP: Au (blue) in the core, and Fe (orange) as the representative element of the PB shell.

The synthesis protocol of (PB)AuNP was monitored by UV–vis spectroscopy (Figure 3A). The modification of the surface of cAuNP to obtain the (PB)AuNP brings changes in the LSPR band position of several nanometers (from 518 to 523 nm). This shift in the wavelength is related to the change in the dielectric constant on the cAuNP surface caused by the PB shell. Concomitantly, a second peak appears at 692 nm. This signal is associated with the absorption of PB, but also with the formation of AuNP dimers within the PB layer.^28,37^ Furthermore, this second peak agrees in position and shape with the UV–vis spectra of the PBNC (maximum at 721 nm), further demonstrating the achievement of the (PB)AuNP synthesis.

Nanomaterial size was obtained by measuring its hydrodynamic diameter (D_H_). At the same time, the success of the modification strategy is evidenced by measuring the Zeta potential (Figure 3B,C, and Table S2). The average D_H_ of cAuNP (29 ± 3 nm) increases after the formation of the shell (69 ± 6 nm). Concerning Z potential, cAuNP presents a negative value of −32 ± 3 mV due to the presence of citrate anions protecting the surface, and it is practically unchanged when these ions are replaced by cyanide from potassium ferrocyanide during the etching step (−35 ± 3 mV) to obtain (CN)AuNP intermediate nanoparticles. Finally, the presence of the PB shell causes a remarkable increase in the negative charge of the modified nanomaterial (−45 ± 5 mV), as expected based on the negative charge of the PB structure itself and the increased compactness of the shell surrounding the particle core after that step.

The size and morphology of the core–shell (PB)AuNP nanostructure were further investigated by transmission microscopy (TEM) (Figure 3D,E). TEM images evidence the typical core–shell structure of the synthesized (PB)AuNP, where the diameter of the core is 18.7 ± 0.6 nm, and the shell size, much irregular, is estimated as 4.2 ± 1.1 nm. EDX elemental mapping indicated that the Au core (blue) is surrounded by a homogeneously distributed PB shell, as evidenced by the mapping of Fe (orange).

Intermediate-nanoparticle TEM micrographics and measured average sizes as well as SEM micrographs corresponding to (PB)AuNP ink were also carried out (Figure S2 and Table S2). The observed homogeneous structure confirms that the prepared ink completely covers the electrode surface.

### In-Channel Electrochemical Cell Characterization

Then, the electrochemical behavior of the four (PB)AuNP-based electrodes integrated into the 3D_d_-ED_d_ devices was deeply investigated by cyclic voltammetry. First, the electrical behavior exhibited by the electrodes before (PB)AuNP-based ink integration was considered. The as-printed CB_PLA_ electrodes exhibit poor electrochemical performance, so activation strategies must be included.^11^ The recorded voltammograms (Figure S3A) show the improvement in electrical conductivity of the CB_PLA_ printed electrodes achieved by electrochemical activation, which is much better with stencil printing C_ink_ electrodes as expected (Table S3). This trend is indistinguishable for the four working electrodes, as maximum peak currents (*I*_c_ = −177 ± 6 μA, *I*_a_ = 185 ± 6 μA), as well as the peak-to-peak separation values (Δ*E*_p_ = 0.133 ± 0.009 V) (*n* = 12) are analogous, demonstrating that the electrochemical behavior is, in the first instance, fully comparable.

Second, the electrical behavior exhibited by the (PB)AuNP-based electrodes was also considered. It is worth noting that the (PB)AuNP/C_ink_/CB_PLA_ indistinctly electrochemical response was observed not only in the two working electrodes within each channel but also in both channels of the 3D_d_-ED_d_ (Figure S3B). The cyclic voltammograms demonstrated the equivalence of the surfaces but also that the electrical conductivity is preserved after this modification, as evidenced by the charge calculated from the integration of the oxidation peak from the PW/PB redox pair (Table S4). Hence, the electrochemical performance of the four working electrodes integrated into the 3D_d_-ED_d_ is equivalent in both as-printed filaments activated as transducers before and after PB modification.

It is also important to note that the (PB)AuNP/C_ink_/CB_PLA_ electrode shows a rough surface, and such roughness affects the diffusion coefficient of electroactive species due to the layering of PB within deposited ink and generally influences the electrochemical responses. The roughness factor, 1.7 ± 0.2, is estimated by cyclic voltammetry, measuring the amount of surface oxide formed by the integration of the gold oxide reduction peak in a cathodic scan (see the Supporting Information for details).^36^ However, this surface roughness given by the ink is not excessively variable between electrodes (RSD ≤ 9%, *n* = 5), a value that proves the proposed assembly method’s efficiency and reproducibility.

### 3D_d_-ED_d_: Analytical Performance

After demonstrating the identical electrochemical behavior of the two pairs of electrodes integrated in series in each channel, the analytical performance of 3D_d_-ED_d_ was also studied toward the detection of H_2_O_2_ at *E*_ap_ = −0.1 V under optimized hydrodynamic flow (500 μL·min^–1^) in 0.1 M DMEM/PBS (pH 7.4) (see Figure S4 for optimizations). In the presence of H_2_O_2_, the reduction peak current increased while the oxidation peak current decreased until it disappeared, evidencing the electroreduction of H_2_O_2_

**Figure 4:**
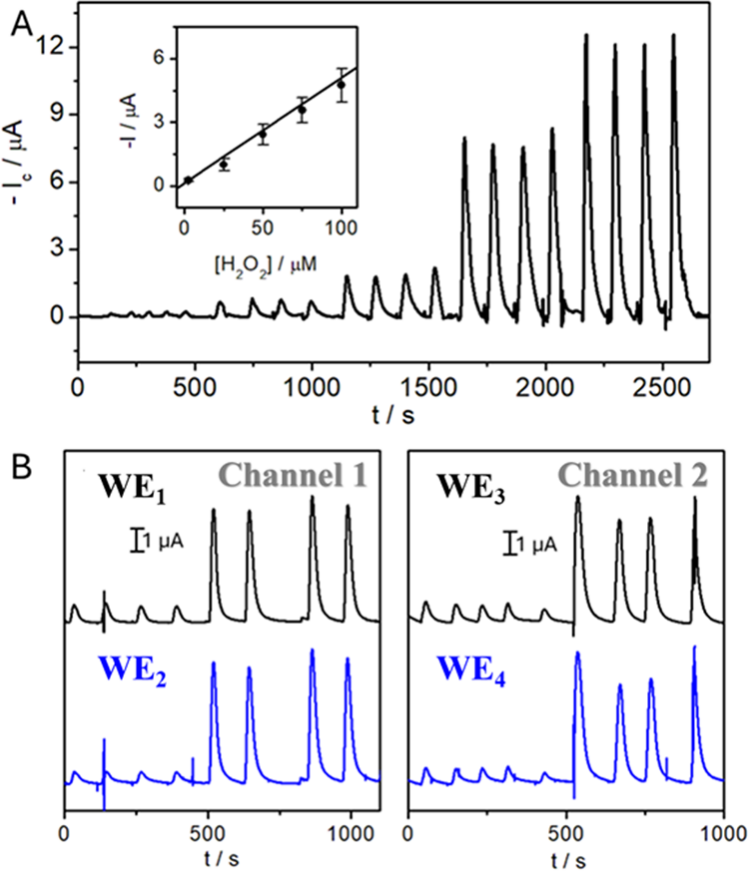
(A) Calibration of H_2_O_2_ in 0.1 M DMEM/PBS recorded under flow conditions (500 μL·min^–1^, *E*_ap_ = −0.1 V, [H_2_O_2_] = 2.5, 25, 50, 75, and 100 μM). Inset: calibration plot (mean values ± standard deviation (*n* = 4 each)). (B) Simultaneous detection in WE_1_^ch1^ and WE_2_^ch1^ of [H_2_O_2_] = 10 and 100 μM. The experiment was sequentially performed in Channel 2 (WE_3_^ch2^ and WE_4_^ch2^).

Excellent analytical performance was obtained with very good linearity (regression equation I (μA) = (0.13 ± 0.03) + (0.050 ± 0.004) [H_2_O_2_], *R*^2^ = 0.995) in the concentration range 2.0–1000 μM, with limits of detection (LOD) and quantification (LOQ) of 0.4 and 2.0 μM, respectively. LOD and LOQ are estimated as LOD = 3*S*_a_/b and LOQ = 10*S*_a_/*b*, where *S*_a_ is the standard deviation of the intercept a and b is the calibration slope. Figure 4A shows the diagrams corresponding to selected hydrogen peroxide concentrations from the working linear range (inset calibration plot, [H_2_O_2_] = 2.5, 25, 50, 75, 100 μM).

The design of the 3D_d_-ED_d_, integrating two working electrodes inside each channel, also results in accurately duplicate data from each experiment, as exemplified in Figure 4B. Currents recorded in the two working electrodes in Channel 1 (−0.63 ± 0.03 μA for [H_2_O_2_] = 10 μM, −4.65 ± 0.46 μA for [H_2_O_2_] = 100 μM) were almost identical to the observed in Channel 2 (−0.62 ± 0.06 μA for [H_2_O_2_] = 10 μM, −4.70 ± 0.24 μA for [H_2_O_2_] = 100 μM, *n* = 4 in all cases).

**Figure 5:**
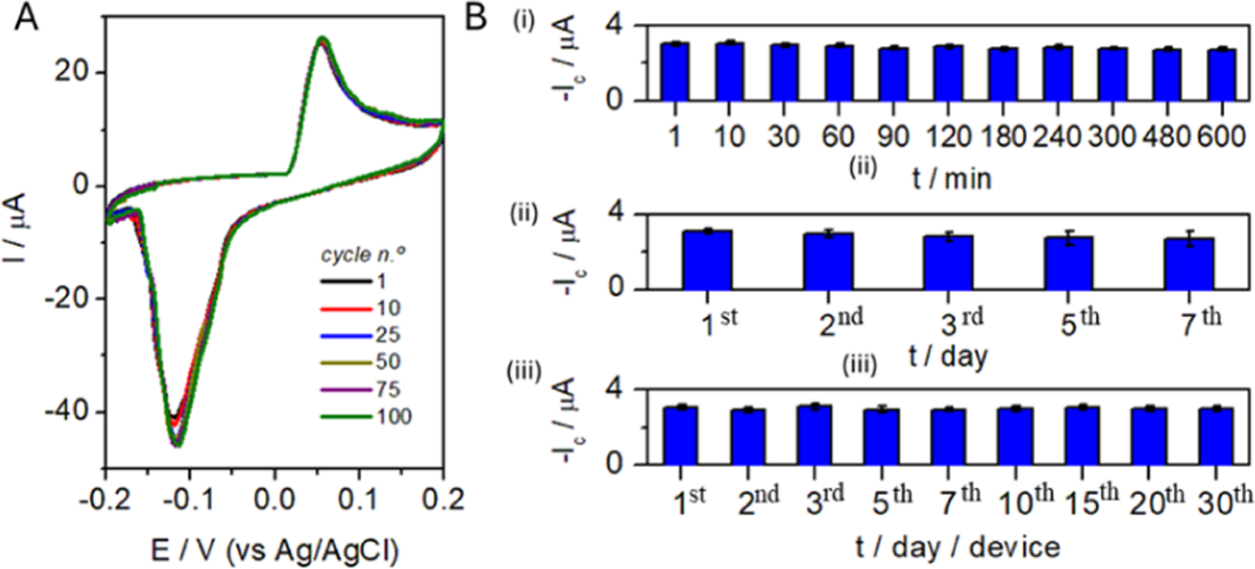
(A) PB(AuNP) stability evaluation by using cyclic voltammetry during 100 cycles (0.1 M DMEM/PBS (pH 7.4), scan rate: 50 mV·s^–1^). (B) 3D_d_-ED_d_ precision evaluation: (i) intraday repeatability assays (same device *n* = 11), (ii) interday intermediate precision assays (*n* = 5 days, same device), and (iii) interdevice intermediate precision assays (*n* = 9 devices and days). Measurements were performed in 0.1 M DMEM/PBS (pH 7.4) by injecting 5 μL of 50 μM H_2_O_2_ aliquots. Values are expressed as mean values ± standard deviation (graphic bars) (*n* = 3 each).

Regarding long-term continuous monitoring, PB layers for sensing applications present a well-known drawback: their intrinsic instability, especially in alkaline conditions due to the interaction between ferric and hydroxide ions to form Fe(OH)_3_, which destroys the Fe–CN–Fe bond, leading to PB solubilization.^38^ Our proposed PB(AuNP) ink solves this problem simply, efficiently, and smartly. Impressively, the current PB(AuNP)/C_ink_/CB_PLA_ stability response did not decrease significantly upon consecutive scans (RSD_i_ < 5%, *n* = 100 cycles) even at physiological pH = 7.4, where iron hydrolysis was negligible, as shown in Figure 5A. The maximum current is reached from the 15th cycle onward, and this behavior can be attributed to the presence of ions trapped in the structure that are released during the first cycles.^39^ This high stability might be due to the negative nature of (PB)AuNP, as evidenced by zeta potential measurements, and the three-dimensional matrix of the electrode surface, which could help reduce the loss of Fe^3+^ due to electrostatic stabilization^40^ Based on the above, several PB(AuNP) sensors were cycled 15 times before evaluating their electrocatalytic performance (*n* = 5 devices), but no significant differences were observed in their response toward H_2_O_2_ detection. The excellent performance could be related to the large specific surface area of (PB)AuNP ink, which speeds up the electron transfer in the sensing process; thus, the detection performance is enhanced.

Then, precision was deeply evaluated in terms of repeatability (intraday assays) and intermediate precision (interday and interdevices assays). The results were excellent (Figure 5B, see also Table S5 for details). The device preserved its integrity for more than 10 h (Figure 5B(i)), effectively detecting hydrogen peroxide (*I*_c_ = −2.98 ± 0.06 μA, RSD ≤ 2% (*n* = 11)). This excellent repeatability encouraged us to explore intermediate precision, on different days, using the same device (Figure 5B(ii)). A current decrease of 10% was found after five measurements; however, analytical parameters are still good (*I*_c_ = −2.76 ± 0.09 μA, RSD_i_ ≤ 8% (*n* = 5 days)). Finally, the interdevice intermediate precision was also explored (*I*_c_ = −2.86 ± 0.07 μA providing an RSD_i_ ≤ 4% (*n* = 9 devices)), performing these assays on different days as well, demonstrating the excellent preservation of the 3D_d_-ED_d_ integrity (Figure 5B(iii)), which we attribute both to the manufacturing protocol and the outstanding properties of the (PB)AuNP transducer. All of these results were obtained for both concentration levels tested, irrespective of the channel and the electrode used, which underlined the reliability of the device.

Finally, potential electroactive interferents such as cysteine, methionine, uric acid, and ascorbic acid were also tested (Table S6 and Figure S5). Intensity changes negligibly at the interferent injection, whereas the expected current is observed by adding H_2_O_2_ (control).

### Reactive Oxygen Species Determination from Caco-2 Cell Culture

The applicability of 3D_d_-ED_d_ was assessed by measuring the amount of H_2_O_2_ released from Caco-2 cells. First, the amount of hydrogen peroxide detected in the culture medium until reaching the confluent state was measured (Figure S6A).

**Figure 6:**
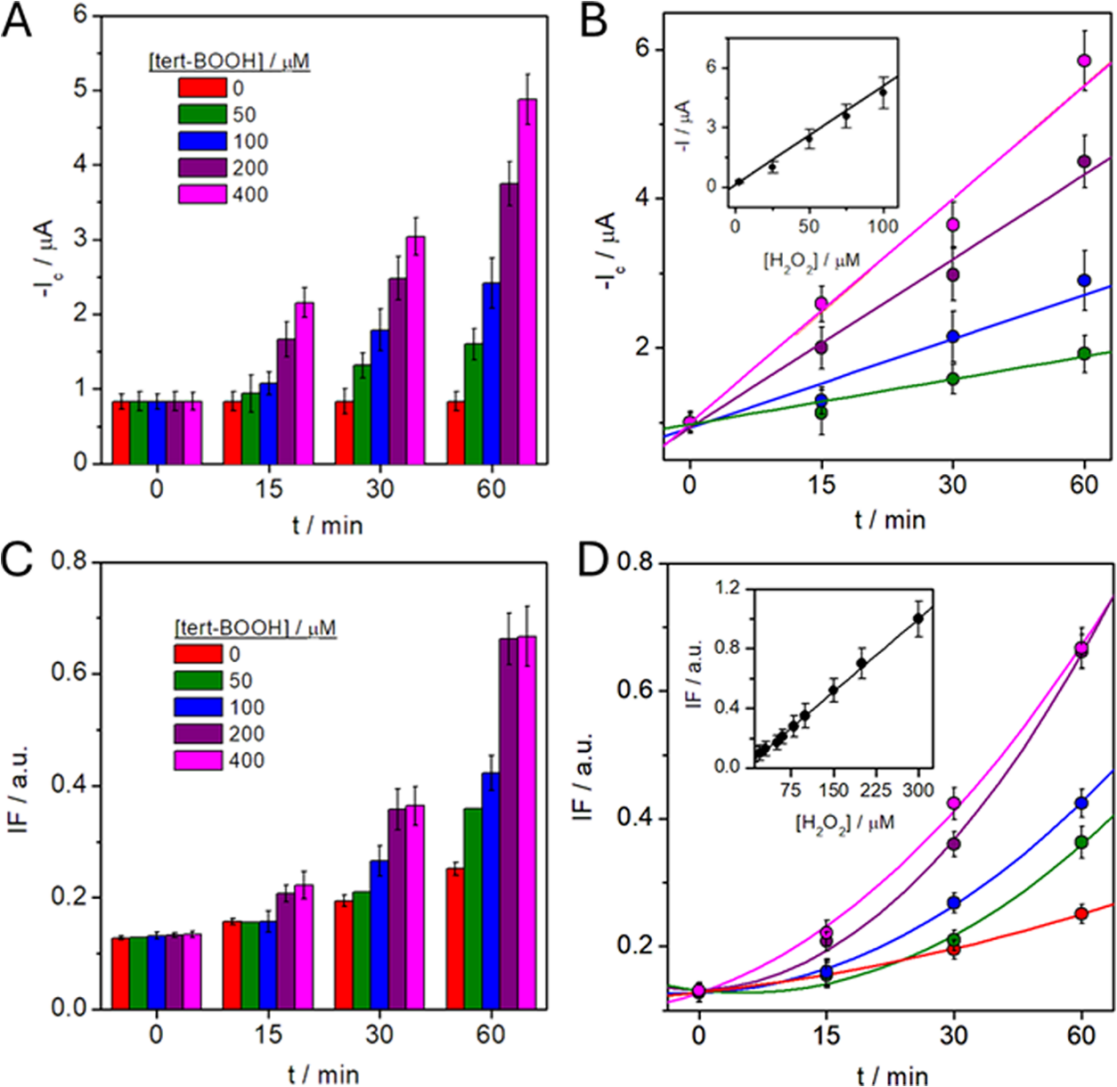
Assessment of ROS from Caco-2 cell cultures released after oxidatively stressed by *t*-BOOH concentrations monitored as a function of the interaction time. (A) Amperometry currents of H_2_O_2_ and (B) relationship between amperometric currents and interaction time, in the presence of several prooxidant concentrations ([*t*-BOOH] = 50, 100, 200, and 400 μM). Inset: calibration plot in the employed concentration range at 60 min. Conditions: 0.1 M DMEM/PBS (pH 7.4), *E*_ap_ = −0.1 V, 500 μL·min^–1^. Fluorescence detection of ROS: (C) fluorescence intensity measurements and (D) relationship between fluorescence intensity and interaction time, using the selected prooxidant concentrations. Inset: H_2_O_2_ fluorescence calibration to determine the ROS levels as H_2_O_2_ equivalents at 60 min. Conditions: 0.1 M DMEM/PBS (pH 7.4), λ_exc_ = 485 nm and λ_em_ = 530 nm Values are expressed as mean values ± standard deviation (graphic bars, *n* = 3 each; calibration plots, *n* = 4 each).

Compared to the control, there is an increase in the current percentage of 14, 27, and 34% during the first 3 days. On the fourth day, the current increase reaches 45% and is maintained from that day forward (*I*_c_ = −0.38 ± 0.02 μA, RSD ≤ 5% (*n* = 3)). Quantitative H_2_O_2_ concentrations were obtained, and they are gathered in Table S7. The above experiment shows that the amount of hydrogen peroxide naturally produced at different levels of growth is not elevated ([H_2_O_2_] = 4.6 ± 0.2 μM, day seventh), which leads us to stimulate the cell cultures. We resorted to two strategies: (i) introduce *E. coli* bacteria into the culture medium and (ii) employ *t*-BOOH as a prooxidant. The effect of *E. coli* stimulation is presented in Figure S6B. Caco-2 cells were exposed to a fixed concentration of bacteria.^41^ Using *E. coli* as an oxidation promoter, the recorded intensity was twice that of the control. The intensity increased over time until it reached its maximum from 3 h and up (*I*_c_ = −1.95 ± 0.14 μA, RSD ≤ 7% (*n* = 3)) ([H_2_O_2_] = 36 ± 1 μM). Finally, we tested the impact of a prooxidant, *t*-BOOH. Cells were exposed to [*t*-BOOH] = 50, 100, 200, and 400 μM, and H_2_O_2_ production was evaluated after 15, 30, and 60 min. The electrochemical response indicates a proportionality between current intensity and interaction time at the explored prooxidant concentrations (Figure 6A,B), reaching a maximum of [H_2_O_2_] = 78 ± 5 μM ([*t*-BOOH] = 400 μM, 60 min). To demonstrate the suitability of the proposed 3D_d_-ED_d_ as a trustworthy PB-based sensor for continuous and selective hydrogen peroxide determination in a cellular medium, we also carried out the analysis by fluorescence. Interestingly, we state that no significant differences between the two approaches over time in the absence and presence of prooxidants were observed (Figure 6C). Indeed, in both approaches, H_2_O_2_ and ROS levels increase with time and prooxidant concentration. However, there is no linear growth but rather a slight exponential trend regarding fluorescence measurements, which we relate to the fact that we are detecting all of the ROS produced, in a nonselective manner (Figure 6D). On that basis, we performed a H_2_O_2_ fluorescence calibration (in fresh cell medium) to determine the ROS levels as H_2_O_2_ equivalents (Figure 6D, inset). H_2_O_2_ levels obtained by electrochemical and fluorescence by using different prooxidant concentrations (see Table S8), exhibited a good correlation value (*r* > 0.90) at low prooxidant levels, being excellent (*r* > 0.999) at the highest ones (above 50 μM) probably due to that the highest amount of ROS generated is transformed into H_2_O_2_ levels at high prooxidant levels. These results highlighted the potential of 3D_d_-ED_d_ in the measurement of H_2_O_2_ levels with long-term stability at physiological pH values.

## Conclusions

We have successfully designed 3D flow-through miniaturized devices integrating a detection cell through two channels, yielding two working electrodes in each, adopting an in-channel configuration. The proposed approach smartly combines the FDM technology with the rich PB-based electrochemistry to obtain ready-to-use 3D-printed devices with excellent analytical performance for hydrogen peroxide detection and determination in cultivar cells. The electrodes exhibited excellent and identical electrochemical performance in both intra- and interchannel, and consequently, they can be used interchangeably in a sequential manner, demonstrating that the 3D technology presents enormous potential in the design of more sophisticated electrochemical (bio)sensing approaches. It also opens the potential use of up to several working electrodes in channels in which independent analytical operations (e.g., calibration) or multiplexed detection could be carried out.

The impress stability of 3D_d_-ED_d_ devices reveals their high potential for real-time monitoring of H_2_O_2_ and other analytes in complex biological systems such as cells/tissues/organoids. Thus, the employment of *ad hoc* 3D printed-based flow devices for real-time measurements, especially when coupled with biological modules, is also envisioned. This incredible stability is intimately related to PB(AuNP)-based electrochemical sensors, which have never been integrated into flow-through devices.

These results are also hopeful since it has demonstrated that the limited resolution of the FMD technology is not a drawback for generating a novel generation of miniaturized electrochemical flow devices with high analytical capabilities, drawing a road toward an independence of the cleanroom facilities and consequently the democratization of the lab on a chip technology in common laboratories.

## Supporting Information Available

Experimental information about alternative PB synthesis protocols, *E. coli* growth conditions, and printing details; extra details related to the electrochemical performance of the dual 3D_d_-ED_d_; (PB)AuNP physical characterization; its electroanalytical performance; and quantitative H_2_O_2_ levels in cell culture studies (PDF)
