# Supplementary material for: 3D-Printed Dual-Channel Flow-Through Miniaturized Devices with Dual In-Channel Electrochemical Detection
Source: Anal Chem. 2024 Dec 24;97(5):2667–77. doi: 10.1021/acs.analchem.4c04099 (PMC11822736; doi:10.1021/acs.analchem.4c04099)
Supplement: Supplementary file 1 — ac4c04099_si_001.pdf [file ac4c04099_si_001.pdf]

Supporting Information

3D printed dual-channel flow-through miniaturized devices with dual in-channel electrochemical detection

Miriam Chávez*[a] and Alberto Escarpa*[a, b]

[a] *Department of Analytical Chemistry, Physical Chemistry and Chemical Engineering, University of Alcalá, E-28805 Madrid, Spain*

[b] *Chemical Engineering and Chemical Research Institute "Andres M. Del Rio", Universidad de Alcalá, E-28802 Madrid, Spain*

Corresponding Authors email: alberto.escarpa@uah.es, miriam.chavez@uah.es

Table of contents

1. Experimental section 2

1.1. Printing details 2

1.2. Complementary Prussian Blue preparation 2

1.2.1. Prussian Blue NanoSpheres and NanoCubes 2

1.2.2. Chemical Prussian blue deposition 3

1.3. E. coli cell culture growth conditions 3

2. Results 3

2.1. Design and Fabrication of the $3D_d$-ED_d devices 3

2.2. Electrochemical Performance of the PB-based electrodes 4

2.3. (PB)AuNP characterization 4

2.4. In-channel electrochemical cell characterization 5

2.4. Analytical performance: definition of the experimental conditions 6

2.4. H_2O_2 levels detected in Caco-2 cells 9

3. References 11

1. Experimental section

1.1. Printing details

EMDs were printed using the following conditions: 0.2 mm layer height, 100% infill percentage, 90° infill orientation, 1.1 extrusion multiplier, 230°C nozzle temperature, 90°C bed temperature, and 25 mm/s print speed. The printing is performed using a 0.4 mm nozzle.

PETg and CB_{PLA} filaments easily absorb moisture. Hydration of the filaments can cause failures during the printing process, and even their deterioration, rendering them unusable. To avoid that, filaments were kept in a filament dryer at 45°C during printing, and at least 2h before it. Filaments were manually changed during the device fabrication. Due to the presence of CB microparticles within the CB_{PLA} filament, a cold pull was performed to remove the excess of this filament from the nozzle after use. This cleaning strategy is necessary both for the cleaning and correct running of the extruder, extending its lifetime, and avoiding the presence of conductive (micro)particles in the non-conductive cover of the device. This cleaning protocol, known as the cold pull procedure, involves the use of a cleaning filament (from SmartMaterials, Spain), and has been previously detailed.[1]

The as-printed devices were activated by the chemical/electrochemical protocol previously reported, in which the channels are first filled with 0.5M NaOH solution, and then fifteen cyclic voltammograms were recorded to measure the electrochemical activation process (-1.0 V to +1.5 V, 0.1 $V \cdot s^{-1}$ scan rate). After that, the channels were immediately washed with water, to remove any products of PLA degradation, and carefully dried with a nitrogen stream, to prevent deterioration.

1.2. Complementary Prussian Blue preparation

1.2.1. Prussian Blue NanoSpheres and NanoCubes

PBNS was prepared by adapting the procedure reported by Maeng et al.[2] 50 mL of 0.5 M HCl solution was placed in a flask (100 mL), and then PVP (40000 MW, 3.8 g) and $K_4Fe(CN)_6 \cdot 3H_2O$ (0.11 g) were added. The mix was sonicated in a water bath for 10 min and then was sealed and stirred for 10 min with a magnetic stirrer at room temperature. Afterward, half of the flask content was transferred to a Teflon autoclave (30 mL capacity) and heated at 80°C for 2h. After that time, a blue precipitate (PBNS) was observed, and it was centrifuged at 8000 rpm and washed three times with distilled water, acetone, and absolute ethanol. Finally, the clean precipitate was fully dried under a vacuum overnight at 35°C.

On the other side, PBNC was synthesized following the protocol employed by X.Shen et al.[3] PVP (40000 MW, 1.5 mg) was dissolved in 20 mL of miliQ water inside a Teflon autoclave (30 mL capacity). The solution was acidified with HCl to pH 2.0 under vigorous stirring for 5 min and, after that, $K_3[Fe(CN)_6]$ (45 mg, 0.136 mmol) was added to the solution. Once it was completely dissolved, the autoclave was sealed and heated to 80°C for 2h. The colloidal suspension was washed by centrifugation (14000 rpm) three times with EtOH and another three with water and finally dried under vacuum overnight at 50°C.

To prepare the PB-based inks, in both cases, the samples are sprayed and weighed, and then the protocol described for (PB)AuNP ink is followed.

1.2.2. Chemical Prussian blue deposition

The modification of the carbon ink surface of the working electrodes with chemically-obtained PB was performed adapting the classical protocol described by Ricci et al.[4] The first layers of the EMD are printed and, at the pause step, after preparing the first layer with carbon ink, a drop of 8 µL of a solution containing 4 µL 0.1 mol·L^{-1} $K_3[Fe(CN)_6]$ in 10 mmol·L^{-1} HCl + 4 µL 0.1 mol·L^{-1} $FeCl_3$ in 10 mmol·L^{-1} HCl is dropped on the working electrode surface. The solution is left on the surface of the electrode for 30 min, maintaining the temperature of the printing bed at 60ºC. After that, it was carefully removed with a micropipette and rinsed with a few millilitres of 10 mmol·L^{-1} HCl. The high solubility of the prepared structure leads us to include a thin film of Nafion (0.1% ethanolic solution) to ensure the integrity of the platform during the experimental measurements. Finally, the printing process restarts until the device is closed.

1.3. E. coli cell culture growth conditions

E. coli cell culture was grown in a 50 mL conical tube (overnight, at 37°, under mild agitation, in Luria-Bertani medium). After that time, the bacteria were washed three times. To do that, 1 mL was centrifugated (1100 RCF, 5 min), the supernatant was discarded, and the bacteria pellet was resuspended in the same volume of Mili-Q water. The concentration of colony-forming units (CFU·mL^{-1}) can be estimated by measuring OD_{600} (with a microplate reader) and applying the following equation:

$$CFU \cdot mL^{-1} = (OD_{600}{}^{4} \cdot 10^{8}) + (2.18 \cdot 10^{7}) \qquad \textbf{(1)}$$

$6.7 \cdot 10^4$ was selected as the *E. coli* concentration employed in the assays as it is a physiologically accurate concentration.[5]

2. Results

2.1. Design and fabrication of the $3D_d$-ED_d devices

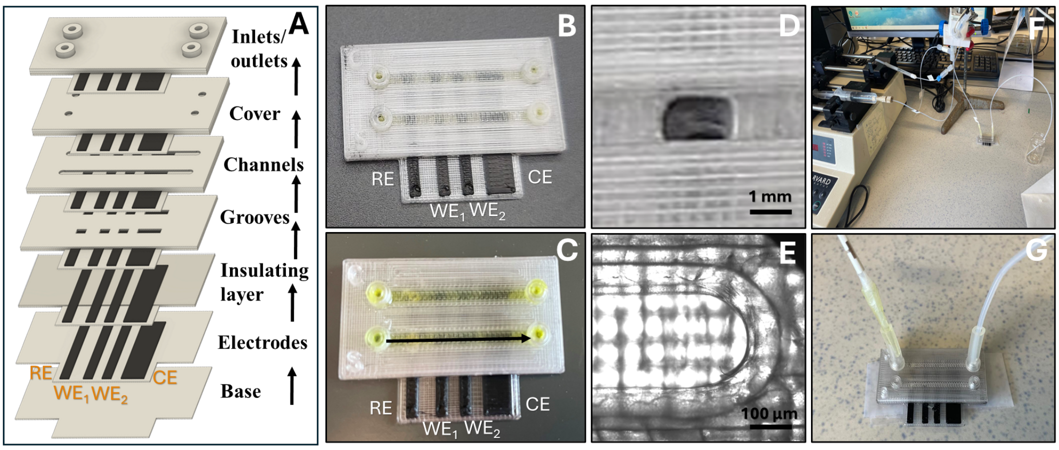

Figure S1. (A) $3D_d$-ED_d design and fabrication steps. Photos of the $3D_d$-ED_d: (B) final device and (C) final device with the channels filled with 0.1 M $[Fe(CN)_6]^{3-}$ + 0.1 M KCl solution. Arrow in C indicates flow direction. This photo was taken one hour after filling, demonstrating no appreciable leakage or deterioration of the device. Magnified photos (taken with a high-performance optical microscope): (D) details of groove/electrodes, and (E) channels. Experimental setup: (F) full view, (G) closer view on the device. Videos illustrating (1) the fabrication of the device (https://youtu.be/LfNeXXTTTCQ) and (2) how the system works (https://youtu.be/Bgx5qOSr5RE) have been recorded.

2.2. Electrochemical Performance of the PB-based electrodes

Table S1. Electrochemical parameters obtained from the analysis of the PW/PB cyclic voltammograms of CB_{PLA}/C_{ink}/PB-based ink working electrodes.

Electrochemical Parameter	PB-structure			
	$PB_{chemical}$	PBNS	PBNC	(PB)AuNP
**E_{pa} / V**	0.032±0.003	0.17±0.01	0.16±0.01	0.027±0.003
**Q_a / µC**	9.1±0.6	26.0±1.0	26.1±0.8	20.5±1.0
**w_a / V**	0.035±0.003	0.150±0.013	0.149±0.008	0.022±0.001
**I_a / µA**	1.9±0.1	1.5±0.1	1.5±0.1	6.4±0.3
**E_{pc} / V**	-0.052±0.002	-0.077±0.008	-0.069±0.009	-0.050±0.002
**Q_c / µC**	12.5±1.2	25.3±1.5	25.3±1.5	22.4±1.4
**w_c / V**	0.031±0.002	0.140±0.010	0.143±0.007	0.032±0.002
**I_c / µA**	-2.5±0.2	-1.4±0.1	-1.5±0.1	-6.7±0.2
**ΔE_p / V**	0.084±0.005	0.243±0.013	0.234±0.015	0.077±0.004
**$E_{1/2}$ / V**	-0.015±0.001	0.045±0.005	0.048±0.002	-0.012±0.001

Mean Values ± Standard Deviation (n=3 electrodes). Conditions: 0.1M KCl/PBS (pH 6.6), scan rate: 10 $mV \cdot s^{-1}$.

2.3. (PB)AuNP characterization

Table S2. Average diameter (from TEM, D_{TEM}, and DLS, D_H, measurements), and Z potential values for the different steps during (PB)AuNP synthesis.

Parameter	cAuNP	(CN)AuNP	(PB)AuNP
**D_{TEM} / nm**	21±3	18±2	23±5
**D_H / nm**	29±3	27±3	69±6
**Z potential / mV**	-32±3	-35±3	-45±5

Mean Values ± Standard Deviation. D_{TEM} diameters are the main value of fifty nanoparticles. D_H and Z potential were measured in diluted solutions (ca. 1.0 nM) of nanoparticles synthesized in different batches (n=3).

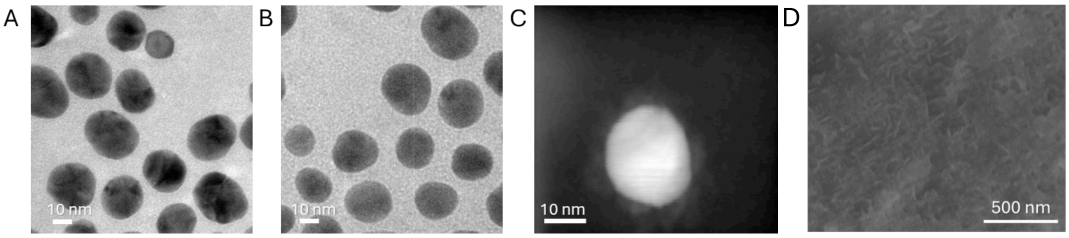

Figure S2. TEM images of (A) cAuNP, and (B) (CN)AuNP. (C) STEM image of (PB)AuNP. (D) SEM image of the $3D_d$-ED_d working electrode surface.

2.4. *In-channel electrochemical cell characterization*

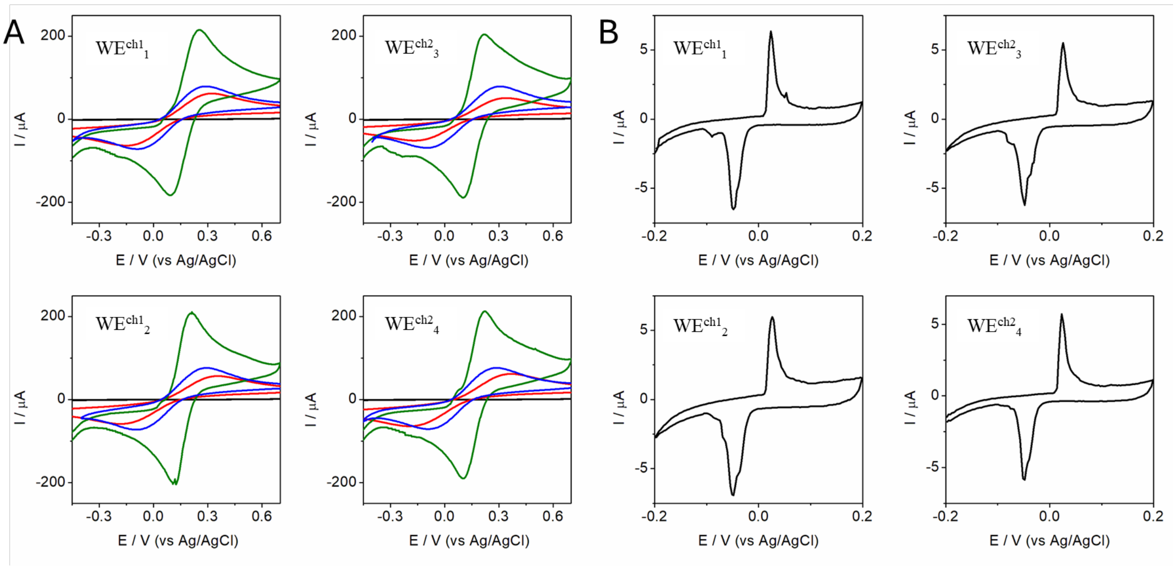

Figure S3. (A) Cyclic voltammograms recorded in the presence of $[Fe(CN)_6]^{3-/4-}$ (5 mM in each component + 0.1M KCl, scan rate: 50 mV·s^{-1}) at the as-printed electrode (black), after electrochemical activation procedure (red), after chemical/electrochemical activation procedure (blue), and with the integration carbon ink electrodes by stencil-printing (green). Activation details (A) (i, red) electrochemical procedure consists of successive 10 scans in 0.5 M NaOH were carried out (0.1 V s^{-1}, from -1.0 V to +1.5 V), while (ii, blue) chemical/electrochemical activation is preceded by the filling of the channel with 0.5 M NaOH solution over 30 min timespan. (B) Electrochemical performance of the four equivalent (PB)AuNP/C_{ink}/CB_{PLA} surfaces in the 3D_d-ED_d evaluated by cyclic voltammetry (0.1M KCl/PBS (pH 6.6), scan rate: 10 mV·s^{-1}).

Table S3. Electrochemical parameters from the analysis of cyclic voltammograms recorded in the presence of $[Fe(CN)_6]^{3-/4-}$ redox probe for the electrochemical activated 3D-printed CB_{PLA} electrodes, and modified with commercial carbon ink.

Electrode	E_{pa} / V	E_{pc} / V	ΔE / V	I_a / μA	I_c / μA
**CB_{PLA}**	0.35±0.02	-0.18±0.02	0.53±0.02	0.059±0.004	-0.064±0.003
**C_{ink}/CB_{PLA}**	0.24±0.02	0.11±0.01	0.13±0.01	0.185±0.006	-0.177±0.006

Mean Values ± Standard Deviation (n=3 devices). Conditions: $[Fe(CN)_6]^{3-/4-}$ (5 mM in each component) + 0.1M KCl, scan rate: 50 mV·s^{-1}.

Table S4. Charge of the oxidation peak (cyclic voltammetry) of the PB/PW couple employed to test the reproducibility of the electrochemical performance of PB-based ink on the $3D_d$-ED_d devices (n=5).

Device number	Channel 1		Channel 2	
	WE_1	WE_2	WE_3	WE_4
1	21.7	23.5	18.8	19.3
2	21.5	20.4	18.3	24.3
3	18.1	19.7	17.9	19.5
4	20.5	18.5	19.8	20.7
5	17.9	22.4	22.0	21.5
Q_a / µC	19.9	20.9	19.3	21.0
SD / µC	1.8	2.0	1.6	2.0
% RSD	9	9	8	9

Conditions: 0.1M KCl/PBS (pH 6.6), scan rate: 10 $mV \cdot s^{-1}$.

Surface roughness

The roughness of the electrode can be estimated by cyclic voltammetry.[6] Oxygen adsorption measurement is a simple in-situ method that indicates the microscopic surface area. It involves oxygen chemisorption onto the gold surface in an anodic potential scan. The amount of surface oxide formed can be measured by integration of the gold oxide reduction peak in a cathodic scan The area under the cathodic peak on the voltammogram is proportional to the real area of the gold surface and is therefore an indication of the surface roughness. The method is based on the electrochemically induced deposition (chemisorption in an anodic potential scan) of an oxygen monolayer on the electrode surface (Au_2O_3) and the measurement of the charge corresponding to this monolayer. By using the following equation,

$$A = \frac{Q \cdot N_A}{n \cdot F \cdot \Gamma} \quad \textbf{(2)}$$

the surface oxide formed can be measured by integrating the gold oxide reduction peak in a cathodic scan (Q). In the equation, N_A is Avogadro's number, F is Faraday's constant, n is the number of electrons involved in the reduction (n=2), and Γ is the theoretical number of Au atoms on the surface ($\Gamma = 1.35 \cdot 10^{15}$ atoms·cm^{-2}).

Based on our measurements, the electroactive area of the electrodes is 5.1±0.3 mm^2, and the geometrical area (from the design) is 3 mm^2. The roughness factor is the relation between these values: 1.7±0.2.

2.4. Analytical performance: definition of the experimental conditions

Evaluation of DMEM/PBS medium

(PB)AuNP/C_{ink}/CB_{PLA} cyclic voltammogram in 0.1M DMEM/PBS (pH 7.4) (50/50% vol) was evaluated (**Figure S4A).** This medium was chosen to determine H_2O_2 as DMEM is widely used in cell cultures and is present in biological samples. Half-wave potential and peak-to-peak separation values are -0.013±0.002 and 0.152±0.08 V, respectively, varying slightly concerning those recorded in 0.1M KCl/PBS. The half-wave potential shift can be directly attributed to the change in the pH of the medium (from 6.6 to 7.4), while the increase in the irreversibility of the voltammogram is also related to the composition of the medium, pointing out the effect on the interfacial electron transfer rate. As the observed differences are minimal, 0.1M DMEM/PBS is considered a suitable medium for H_2O_2 monitoring employing the developed sensor. H_2O_2 detection is first evidenced by cyclic

voltammetry (**Figure S4B).** The reduction peak current increased in the presence of H_2O_2, while the oxidation peak current decreased until it disappeared. The observed behavior evidences the electroreduction of H_2O_2 on the electrode surface by PW,[7] demonstrating the potential for selective H_2O_2 detection in the presence of O_2 of the developed $3D_d$-ED_d.

Selection of the hydrodynamic potential

Furthermore, an adequate potential to perform the measurements was selected. This hydrodynamic potential was chosen after several measurements of 300 µM H_2O_2 in 0.1 M DMEM/PBS solution (**Figure S4C)**. At more cathodic potentials, higher current responses are acquired. At -0.1 V, a significant increase in the current response is obtained, and the application of more cathodic potentials produces only a slight increase of 10% in the current. However, at such potentials, parallel reactions could begin to take place, so interference with other compounds may be considerable, and, more importantly, a higher overpotential should be applied. This led us to choose -0.1 V as the applied hydrodynamic potential (E_{ap}).

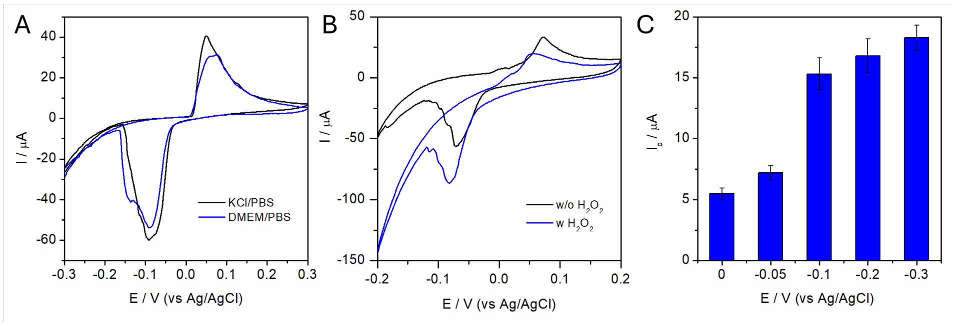

Figure S4. (A) Effect of the media on the cyclic voltammogram of the sensor: 0.1 M DMEM/PBS (pH 6.6) (black) and 0.1M DMEM/PBS (pH 7.4) (blue). (B) Electrocatalytic response of (PB)AuNP/C_{ink}/CB_{PLA} towards H_2O_2 detection ($[H_2O_2]$ = 50 µM, 0.1 M DMEM/PBS (pH 7.4), scan rate: 50 $mV·s^{-1}$). (C) Hydrodynamic potential selection. Conditions: 0.1 M DMEM/PBS (pH 7.4), injections of $[H_2O_2]$ = 300 µM, flow rate: 500 $µL·min^{-1}$. Values are expressed as Mean Values ± Standard Deviation (graphic bars in C) (n=3 each).

Precision studies

Table S5. Precision performance of the four equivalent (PB)AuNP/C_{ink}/CB_{PLA} working electrodes integrated in the $3D_d$-ED_d.

Precision assessment	Channel 1		Channel 2	
	**WE_1**	**WE_2**	**WE_1**	**WE_2**
**Repeatability (n=11)**	-0.62±0.04 (6)	-0.64±0.03 (4)	-0.66±0.05 (8)	-0.58±0.03 (6)
	-4.40±0.4 (8)	-5.0±0.5 (9)	-4.53±0.4 (6)	-4.83±0.4 (4)
**Intermediate precision (n=5 days)**	-0.63±0.03 (5)		-0.62±0.06 (9)	
	-4.70±0.46 (9)		-4.68±0.24 (5)	
**Intermediate precision (n=9 devices)**	-0.62±0.06 (9)			
	-4.83±0.37 (8)			

Values are expressed as Current Intensity Mean Value ± Standard Deviation (μA) (R.S.D., %). Each injection by triplicate. Conditions*:* measurements were performed in 0.1M DMEM/PBS (pH 7.4) by injecting 5 μL of 50 μM of H_2O_2.Two analyte concentrations were chosen: [H_2O_2] = 10 μM (grey rows), [H_2O_2] = 100 μM (white rows).

Interference study

An interference study from common electroactive biomolecules usually present in cellular medium was performed. Specifically, the potential interference of common acidic compounds (uric and ascorbic acids) and amino acids (cysteine and methionine) was explored. Tested concentrations were selected considering their physiological standard values (**Table S6**), and the response was monitored by adding the maximum within the standards, and twice this concentration. **Figure S5** shows that intensity changes negligibly at the interferents injection, whereas the expected current is observed adding 10 μM H_2O_2.

Table S6. Physiological standard values in plasma for the explored interferents.

Compound	Range / mg·L^{-1}	Evaluated values / mg·L^{-1}	Reference
**Uric Acid**	35-72	70 and 140	8
**Ascorbic Acid**	7-14	14 and 30	9
**Cysteine**	20.2 – 28.58	30 and 60	10
**Methionine**	1.81 – 5.74	6 and 12	11

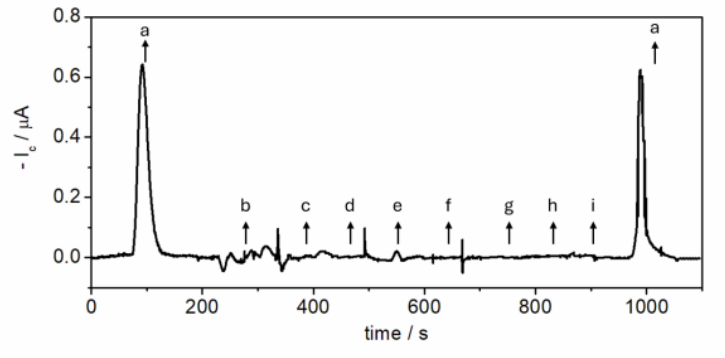

Figure S5. Amperometric response of the sensor to interference substances: (a) H_2O_2 10 µM; uric acid (b) 70 and (c) 140 (mg·mL^{-1}); ascorbic acid (d) 14 and (e) 30 (mg·mL^{-1}); cysteine (f) 30 and (g) 60 (mg·mL^{-1}); methionine (h) 6 and (i) 12 (mg·mL^{-1}). Conditions: 0.1M DMEM/PBS (pH 7.4), E_{ap} = -0.1 V, 500 µL·min^{-1}.

2.4. H_2O_2 levels detected in Caco-2 cells

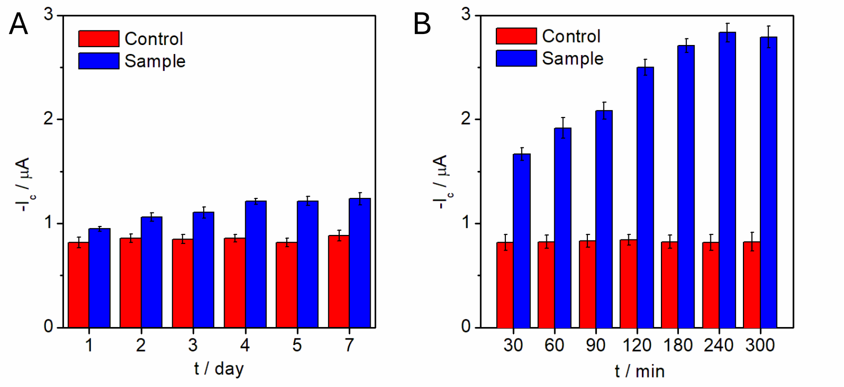

Figure S6. Measurement of H_2O_2 production as ROS indicator from Caco-2 cell culture. Amperometry currents of H_2O_2 released (A) at different days without stimulation, (B) oxidatively stressed by *E. coli*. Values are expressed as Mean Values ± Standard Deviation (graphic bars) (n=3 each).

Table S7. Quantitative levels of H_2O_2 released from Caco-2 cell as a function of evolution time.

Number days	1st	2nd	3th	4 th	5 th	7 th
**$[H_2O_2]$ /µM**	-	-	2.6±0.1	4.9±0.2	5.6±0.2	4.6±0.2

Mean Values ± Standard Deviation (n=3 each).

Table S8. Quantitative levels of H_2O_2 released from Caco-2 cell after being stressed by a fix concentration of *E. coli.*

Time/min	30	60	90	120	180	240	300
**$[H_2O_2]$ /µM**	14±1	19±1	23±1	31±1	35±1	36±1	37±1

Mean Values ± Standard Deviation (n=3).

Table S9. H_2O_2 released from Caco-2 cell after being stressed by several concentrations of t-BOOH, employed as a strong prooxidant.

	**$[H_2O_2]$ /µM**		
**[t-BOOH] /µM**	**15 min**	**30 min**	**60 min**
**50**	-	6.7±0.3	13±1
**100**	1.7±0.2	16±1	31±2
**200**	10±1	30±2	57±3
**400**	24±2	42±3	78±5

Mean Values ± Standard Deviation (n=3).

Table S10. Hydrogen peroxide levels and correlations obtained by electrochemical and fluorescent measurements at different prooxidant concentrations and exposition times.

Time	[t-BOOH] / µM	50	100	200	400
**15 min**	$[H_2O_2]$ (EQ) / µM	0	1.1±0.3	10±1	24±2
	$[H_2O_2]$ (F) / µM	39±2	42±3	57±3	61±5
**30 min**	$[H_2O_2]$ (EQ) / µM	6.7±0.8	13±1	30±2	42±6
	$[H_2O_2]$ (F) / µM	57±3	73±3	103±4	110±9
**60 min**	$[H_2O_2]$ (EQ) / µM	13±1	25±2	57±2	78±3
	$[H_2O_2]$ (F) / µM	103±5	122±3	195±6	198±8
**Correlations (r)**		0.965	0.992	0.995	0.999

EQ: Electrochemical measurements recorded via $3D_d$-ED_d, **F**: fluorescence signals (microplate reader, λ_{exc}=485 nm and λ_{em}=530 nm)

3. References

(1) Hernández-Rodríguez, J. F.; Rojas, D.; Escarpa, A. Electrochemical Fluidic Fused Filament Fabricated Devices (EF^4D): in-channel electrode activation. *Sens. Actuators B Chem.* **2023**, *393*, 134290.

(2) Maeng, H. J.; Kim, D.-H.; Kim, N.-W.; Ruh, H.; Lee, D. K.; Yu, H. Synthesis of spherical Prussian Blue with high surface area using acid etching. *Curr. App. Phys.* **2018**, *18*, S21–S27.

(3) Shen, X.; Wu, S.; Liu, Y.; Wang, K.; Xu, Z.; Liu, W. Morphology syntheses and properties of well-defined Prussian Blue Nanocrystals by a facile solution approach. *J. Colloid Interface Sci.* **2009**, *329* (1), 188–195.

(4) Ricci, F.; Amine, A.; Palleschi, G.; Moscone, D. Prussian Blue based screen-printed biosensors with improved characteristics of long-term lifetime and pH stability. *Biosens. Bioelectron.* **2003**, *18* (2–3), 165-174.

(5) Luchan, J.; Choi, C.; Carrier, R. L. Reactive oxygen species limit intestinal mucosa-bacteria homeostasis in vitro. *Sci. Rep.* **2021**, *11* (1).

(6) Hoogvliet, J.C.; Dijksma, M.; Kamp, B.; Van Bennekom, W. P. Electrochemical pretreatment of polycrystalline gold electrodes to produce a reproducible surface roughness for self-assembly: a study in phosphate buffer pH 7.4. *Anal. Chem.* **2000**, 72 (9), 2016-2021.

(7) Karyakin, A. A.; Karyakina, E. E.; Gorton, L. On the mechanism of H_2O_2 reduction at Prussian Blue modified electrodes. *Electrochem. Commun*. **1999**, *1* (2), 78–82.

(8) Kandár, R.; Záková, P.; Muzáková, V. Monitoring of antioxidant properties of uric acid in humans for a consideration measuring of levels of allantoin in plasma by liquid chromatography. *Clin. Chim. Acta* **2006**, *365* (1–2), 249–256.

(9) Chung, W. Y.; Chung, J. K. O.; Szeto, Y. T.; Tomlinson, B.; Benzie, I. F. F. Plasma ascorbic acid: measurement, stability, and clinical utility revisited. *Clin. Biochem* .**2001**, *34* (8), 623–627.

(10) Pastore, A.; Alisi, A.; di Giovamberardino, G.; Crudele, A.; Ceccarelli, S.; Panera, N.; Dionisi-Vici, C.; Nobili, V. Plasma levels of homocysteine and cysteine increased in pediatric NAFLD and strongly correlated with severity of liver damage. *Int. J. Mol. Sci.* **2014**, *15* (11), 21202–21214.

(11) da Costa, K. A.; Gaffney, C. E.; Fischer, L. M.; Zeisel, S. H. Choline deficiency in mice and humans is associated with increased plasma homocysteine concentration after a methionine load. *Am. J. Clin. Nutr.* **2005**, *81* (2), 440–444.
